# Numerical Analysis of Incinerator Refractory Brick with Coupled Parameters Based on Thermodynamic Theory

**DOI:** 10.3390/ma18040824

**Published:** 2025-02-13

**Authors:** Yu Mou, Sisi Han, Yanrong Zhang, Kai Wu, Xinrui Shen

**Affiliations:** 1 Key Laboratory of Cremation Equipment, 101 Institute of The Ministry of Civil Affairs, Beijing 100070, China; 13810898296@139.com; 2 School of Civil Engineering, Beijing Jiaotong University, Beijing 100044, China; 20115060@bjtu.edu.cn (K.W.); 23121204@bjtu.edu.cn (X.S.)

**Keywords:** refractory brick, incinerator, numerical simulation, coupled thermal stress, material parameters, structural parameters

## Abstract

The selection of refractory bricks significantly impacts the operational performance of brick structures in high-temperature environments. In this study, a coupled thermal stress model of a refractory brick structure was established and validated by means of thermal expansion experiments. This paper innovatively combined the brick number, brick thickness, and brick material to investigate their influence on brick structural performance. The results indicated that the influence of the brick number on the temperature was less significant than that of brick thickness. However, the brick number had a greater effect on vertical displacement and principal compressive stress than brick thickness, with the maximum differences being 342.3% and 28.9%. Compared to brick thickness, brick material had a more significant effect on vertical displacement and principal compressive stress, with the maximum differences being 77.1% and 67.4%. Additionally, the influence of brick material properties on vertical displacement and principal compressive stress was greater than that of the brick number, with the maximum differences being 77.6% and 65%. Therefore, when selecting refractory bricks, it is advisable to consider the brick material first, the brick number second, and the brick thickness last. This study offers theoretical guidance for refractory brick structure design and material selection in high-temperature applications.

## 1. Introduction

With the continuous advancement of industrial technology, an increasing amount of waste is generated. As a key piece of waste management equipment, incinerators face issues such as concentrated thermal stress [1] and poor thermal shock resistance. Moreover, the choice of refractory bricks significantly affects their operational performance under high-temperature conditions. Therefore, it is crucial to select appropriate refractory bricks to maintain stability and durability under high-temperature conditions [2]. Investigators have employed numerical simulation techniques to delve into the thermal stress distribution, erosion status, and structural optimization [3,4,5] of incinerators. For example, Gan et al. [6] utilized a coupled thermal fluid–solid model to analyze thermal stress in hot blast furnaces. Shi Zifu et al. [7] investigated the impact of the incineration furnace structure on safety and emissions when burning highly calorific waste. Liu Xianrong et al. [8] employed Flic and Fluent to enhance combustion efficiency through exhaust and material design. In order to effectively reduce heat loss on the surface of a rotary kiln, Chu Linhua [9] optimised the structure of refractory bricks using finite element simulation technology. Wang Zai [10] employed phase field methods to simulate crack propagation in refractory materials. Ramaneka D [11] assessed refractory materials’ service lives and resistance to slag through simulations. Yalan Yu [12] developed chromium-free refractory materials and furnace lining designs for coal catalytic gasification furnaces to improve their erosion resistance. Thermo-mechanical coupling in incineration furnaces, particularly between refractory materials and molten slag, has also been studied to determine the temperature and stress field distributions and thermal shock stability. For example, Brulin J [13] demonstrated that thermo-mechanical coupling significantly impacts refractory material damage under high-temperature and complex-stress conditions. D, Gruber [14] simulated the internal temperature distribution of refractory materials. Chartier et al. [15] applied CFD in 2007 to understand the waste incineration process and proposed optimizations for furnace design and operation. Lin Hai et al. [16] optimized the combustion and heat transfer processes in waste incineration furnaces for safe operation. Ding Jiahui et al. [17] discussed the types of refractory materials, the damage mechanisms, and the development direction for hazardous waste incineration furnace linings. Wang et al. [18] discussed the heat transfer calculations of different continuous heating furnace lining structures and concluded that the dominant structures were as follows: a 1250 °C steel rolling furnace with a 105 mm fiberboard, 230 mm JM23 insulating brick and 115 mm light mullite firebrick; and a 1600 °C tunnel kiln with a 110 mm fiberboard, 115 mm JM23 insulating brick, 115 mm light mullite firebrick, and 160 alumina bubble brick. Miyazato Tatsushi [19] tested 9-inch bricks and found that their linear shrinkage was less than 1% at 1450 °C and their volume shrinkage was less than 2% at 1600 °C. In order to carry out the work of revising the national standard GB/T 2992-82 “Shape and Size of General Refractory Bricks”, Xue [20] studied the size series of refractory bricks in China. Li [21] designed the shape and size according to the unique properties of silicon carbide products and the actual working scene of the products, which saved resources and prolonged the service life of the furnace lining.

**Figure 1:**
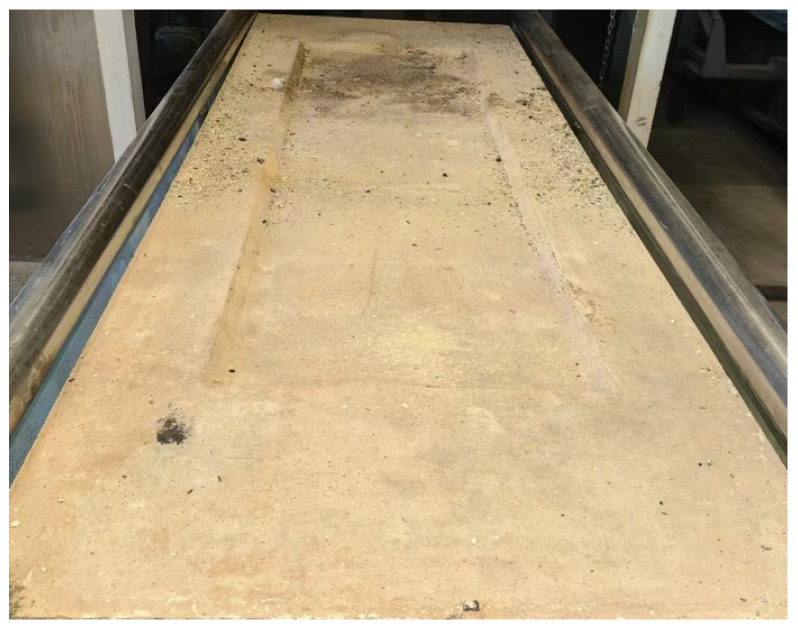
Refractory brick.

Currently, the existing research primarily focuses on the optimization of the microstructural properties of refractory materials and enhancement of the overall structural performance of incinerators, while few studies have conducted coupled analyses of the structural parameters and materials of incineration refractory bricks. In addition, the available numerical simulation studies on refractory bricks have mainly focused on small-sized refractory bricks, while fewer studies have been conducted on large-size refractory bricks. In order to help the reader better understand such large-size refractory bricks, Figure 1 shows a physical diagram of 2.2 m × 0.78 m × 0.1 m corundum–silicon carbide refractory brick (single brick). This study is based on the actual structure of self-developed large-size refractory bricks in incinerators, integrated with actual measured data from the temperature variation process of the incinerator, and it employs a fully coupled thermo-stress method [22] to establish a thermo-stress coupled model of the refractory bricks in the incinerator. This model is then validated through thermal expansion tests. This research innovatively applies the refractory brick model used for the coupling of structural parameters and brick materials to analyze their impact on the structural performance of the refractory bricks, with the expectation of establishing a prioritized sequence of coupled parameters to consider when selecting refractory bricks. This research is expected to provide new references for ensuring the safe and reliable operation of incineration furnaces, as well as for the design, production, and engineering application of long-life refractory brick structures.

## 2. Basic Theory of Coupled Thermodynamic Analysis

In the analysis of engineering structures, objects often simultaneously endure thermal and stress loads. The fully coupled thermo-stress method in finite element methods can simultaneously consider the interaction between the temperature field and the stress field. Therefore, this paper uses fully coupled thermo-mechanical analysis, which can more accurately reflect the actual physical process.

The fully coupled thermo-mechanical formulation in Abaqus is rooted in the principles of continuum thermodynamics, specifically the conservation of energy and the second law of thermodynamics. The governing equations are derived as follows.

Energy conservation—the balance of internal energy in a continuum is expressed as the following:

(1) $$\rho \dot{e}=\sigma :\dot{\varepsilon }-\nabla \cdot q+\rho r$$

where ρ is the material density, e is the specific internal energy, σ is the Cauchy stress tensor, $\dot{\varepsilon }$ is the strain rate tensor, q is the heat flux vector, and r is the volumetric heat source. This equation couples mechanical work $\left(\sigma :\dot{\varepsilon }\right)$ with thermal energy transfer (∇⋅q) [23].

Entropy inequality—the Clausius–Duhem inequality constrains constitutive models to ensure thermodynamic consistency:

(2) $$\rho \dot{s}+\nabla \cdot \left(\frac{q}{T}\right)-\frac{\rho r}{T}\geq 0$$

where s is the specific entropy and T is the absolute temperature [24].

For the linear thermoelastic material, thermoelastic constitutive equations are used:

(3) $$\sigma =C:\left(\varepsilon -\alpha \left(T-T_{0}\right)I\right)$$

where C is the elasticity tensor, α is the coefficient of thermal expansion, and $T_{0}$ is the reference temperature [25].

The temperature evolution is governed by Fourier’s law and the energy balance:

(4) $$\rho c_{p}\dot{T}=\nabla \cdot (k\nabla T)+\sigma :{\dot{\varepsilon }}^{mech}+\rho r$$

where $c_{p}$ is the specific heat at constant pressure, k is the thermal conductivity, and ${\dot{\varepsilon }}^{\mathrm{mech}}$ is the mechanical strain rate. The term $\sigma :{\dot{\varepsilon }}^{\mathrm{mech}}$ accounts for thermoelastic dissipation, representing heat generation due to mechanical work [26].

In Abaqus, the heat conduction equation is commonly discretized using the finite element method. By integrating the heat conduction equation over the elements, the following elemental heat balance equation can be derived [27,28,29]:

(5) [C]{T}+[K]{T}={Q}

where [C] is the heat capacity matrix, [K] is the heat conduction matrix, {T} is the vector of nodal temperatures, and {Q} is the vector of heat sources. Numerical methods are employed to solve this system of equations, yielding the temperature distribution within the object.

The mechanical equilibrium equation is also discretized using the finite element method. By integrating the mechanical equilibrium equation over the elements, the elemental mechanical equilibrium equation is derived [30,31]:

(6) $$\left[K_{u}\right]\{u\}=\{F\}$$

where $\left[K_{u}\right]$ is the stiffness matrix, {u} is the vector of nodal displacements, and {F} is the vector of nodal forces. Then, using a similar approach, the stress and strain distribution within the object is obtained.

Fully coupled thermo-mechanical analyses in ABAQUS are used with thermal and mechanical analyses simultaneously. This method provides a quadratic convergence speed and checks for convergence. If the convergence criteria are not met, the process iterates until the criteria are satisfied.

## 3. Numerical Simulation

### 3.1. Coupled Thermal Stress Model of Incinerator Refractory Bricks

**Figure 2:**
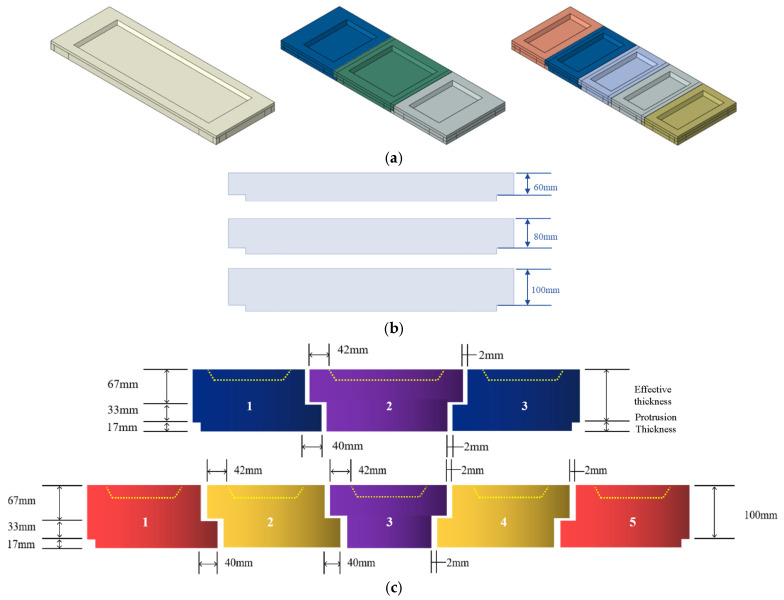
Schematic diagram of the finite element model of refractory bricks. (**a**) Single-brick, three-brick, and five-brick models; (**b**) refractory brick of different thicknesses; and (**c**) splicing of three and five refractory bricks.

In this study, a fully coupled thermal stress approach within Abaqus was used to construct a three-dimensional full-scale model of refractory bricks. Specific input parameters in the FEM of the refractory brick structure is provided in Table 1 [32,33,34,35], with the material parameters used for the finite element model detailed in Table 2. Front views of the refractory bricks with different radial thicknesses are depicted in Figure 2b, while the complete assemblies of single-brick, three-brick, and five-brick models are shown in Figure 2a. Taking the brick thickness of 100 mm as an example, the schematic diagram of the gaps and thickness of the three-brick and five-brick structures is shown in Figure 2c. It can be seen from the figure that the actual thickness was calculated as the sum of the effective thickness and the protrusion thickness. In this study, the brick thickness was used to denote the effective thickness of the brick. Moreover, the overall length of the structure was 2.2 m and the width was 0.78 m. When the brick number was three, the upper surface length of brick no. 2 was 0.8 m, while the upper surface lengths of bricks no. 1 and no. 3 were 0.7 m. The width of all bricks was 0.78 m. When the brick number was five, the upper surface length of each brick was 0.44 m and the width was 0.78 m. Additionally, Figure 2c also illustrates the structural assembly of the three-brick and five-brick models. We noticed that there was a 2 mm gap between all neighboring bricks. For both the three-brick and five-brick structures, a groove with a depth of 30 mm was present on the upper surface of the refractory bricks. For ease of fixation, a protrusion with dimensions of 2.114 m × 0.694 m × 0.017 m was present at the bottom. All models were simulated using C3D8T elements [36], with a primary mesh size of approximately 50 mm, and the mesh in the gap areas was refined to ensure accuracy.

**Figure 3:**
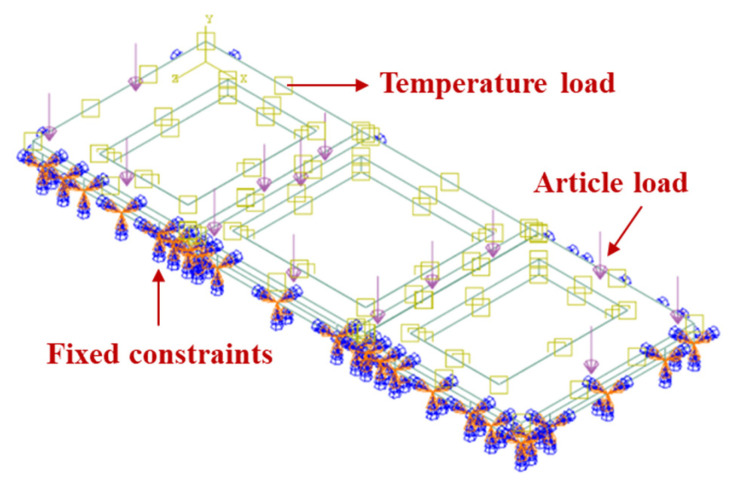
Boundary condition diagram for the three-refractory-brick model.

**Figure 4:**
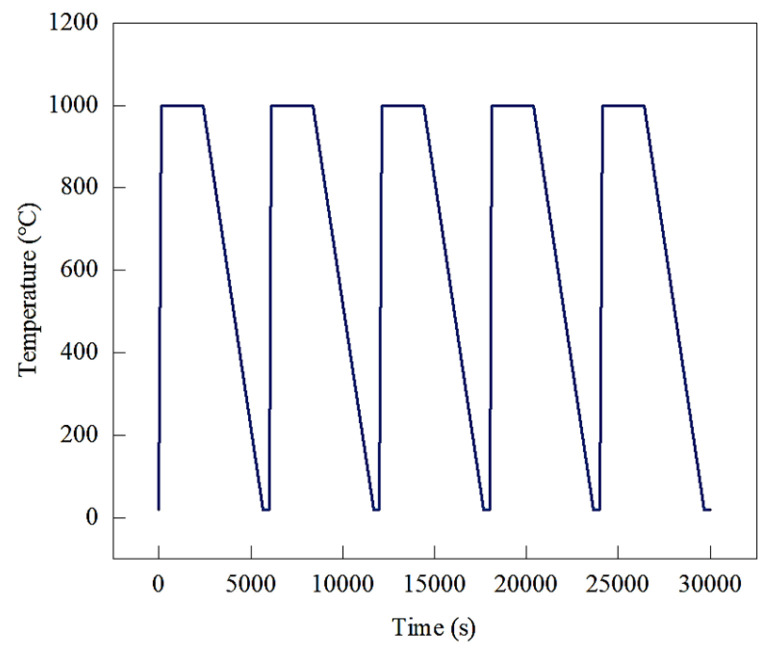
Schematic diagram of the temperature cycles.

For the finite element model of the three refractory bricks, the boundary conditions are illustrated in Figure 3. The upper surface of the refractory bricks was defined as the heat transfer surface, while fixation constraints were applied at the bottom ring of the refractory bricks for a cycle to simulate the actual bottom frame fixation. The model underwent a total of five temperature cycles, each with a duration of 100 min, encompassing heating, isothermal holding, and cooling phases. For a maximum service temperature of 1000 °C, the temperature cycling is depicted in Figure 4. The model was based on measured data; the heating rate was kept at 8.45 °C/s and the cooling rate at 0.30 °C/s. The initial temperature of the whole model was set to 20 °C.

### 3.2. Thermal Expansion Testing of Refractory Materials

**Figure 5:**
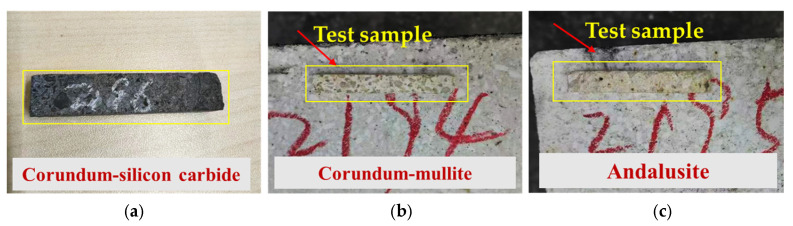
Thermal expansion test samples of refractory materials. (**a**) Corundum–silicon carbide samples; (**b**) corundum–mullite samples; and (**c**) andalusite samples.

Accurately measuring the temperature of a large structure is extremely challenging, so it is difficult to validate the accuracy of refractory brick models through full-scale experiments. To substantiate the reliability and accuracy of the refractory material thermal expansion numerical model established in this section, thermal expansion tests were conducted on scaled samples of corundum–mullite, andalusite, and corundum–silicon carbide, in accordance with the national standard GB/T 7320-2018 [37], by employing the vertical push-rod technique. Figure 5 sequentially displays the dimensions of the corundum silicon carbide samples, corundum–mullite samples, and andalusite samples before thermal expansion testing with dimensions of 7 mm × 7 mm × 50 mm (within the yellow box). Specifically, the experimental test procedure [37] is as follows. Prior to the thermal expansion tests, specimens were prepared from suitable locations at least 15 mm from the edge of the sample bricks, with dimensions of 7 mm × 7 mm × 50 mm, in the form of cuboids. Each specimen was placed within the heating furnace and positioned in the sample compartment, with one end secured and an initial force below 2.0 N applied to the other end. This ensured the stability of the specimens and eliminated any gaps between them. The specimens were heated to the designated test temperature at a rate of 4 °C/min. The change in the length of the specimens from room temperature to the test temperature of 1000 °C was measured, and simultaneous strain collection was carried out. This process yielded the strain test results for the specimens, from which the average linear expansion ratio data were derived.

### 3.3. Thermal Expansion Testing Model for Refractory Brick Specimens

**Figure 6:**
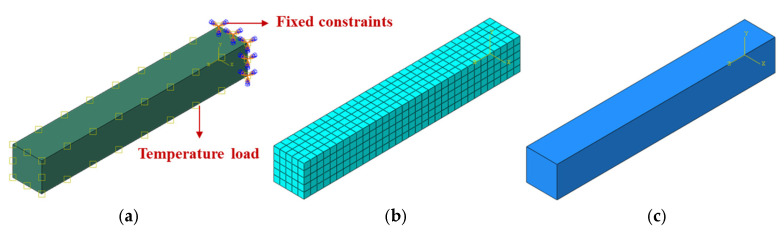
Experimental numerical model of thermal expansion of refractory brick specimens. (**a**) Boundary condition diagram; (**b**) mesh delineation diagram; and (**c**) full 3D view of the model.

A thermo-expansion experimental model (as depicted in Figure 6) was established for the thermal expansion testing of refractory materials. The model comprised cuboidal samples measuring 7 mm × 7 mm × 50 mm. The validation model consisted of 5000 mesh elements and employed C3D8T finite element units, which facilitated the integration of displacement and temperature fields, thereby enabling precise simulations of complex thermo-structural interaction problems. Fixed constraints were applied to one end of the model to simulate the immobilization at the bottom during the testing phase. The initial temperature of the model was defined as room temperature, and the model warming rate was consistent with that described in Section 3.1. The material parameters of the refractory bricks were taken from Table 2, while the linear expansion coefficients were taken from the data measured in the linear expansion tests described in Section 3.2.

**Figure 7:**
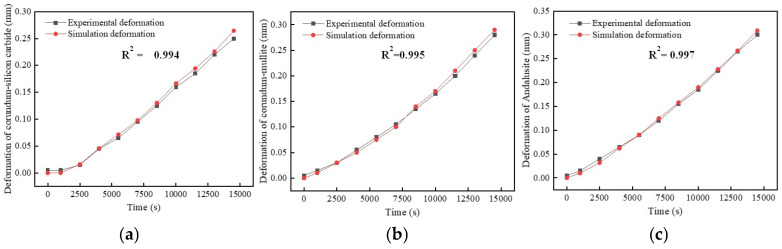
Comparison between model results and experimental results. (**a**) Deformation of corundum–silicon carbide; (**b**) deformation of corundum–mullite; and (**c**) deformation of andalusite.

A comparison of the simulation results obtained from the numerical model and the experimental results for the thermal expansion of the refractory brick samples is shown in Figure 7. It is clear from the figure that the corundum–silicon carbide model, the corundum–mullite model, and the andalusite model produced deformation trends that were comparable to the increasing trends derived from experimental measurements [38], with essentially the same pattern of development. It is worth noting that andalusite refractory bricks in this study have the smallest modulus of elasticity and the smallest coefficient of thermal expansion of the three materials. Furthermore, in order to make the validation experimental model consistent with the experimental conditions for linear expansion, one side of the model was constrained while the other end was free to deform. Hence, the modulus of elasticity plays a decisive role in the deformation of the material, and the final experimental result shows that the andalusite refractory bricks have the maximum deformation. Moreover, the models’ respective decision coefficients (R^2^) between the modelled length variation and the experimental length variation stood at 0.994, 0.995, and 0.997. Thus, it can be proven that the coupled thermal stress model constructed in this study has good validity and accuracy.

## 4. Results and Discussion

### 4.1. Combined Effects of the Brick Number and Brick Thickness

This section analyzes the six scenarios depicted in Table 3 to explore the combined impact of different quantities and thicknesses of corundum–silicon carbide refractory bricks on their temperature distribution, stress state, and deformation at an elevated temperature of 1000 °C.

#### 4.1.1. Temperature

During the incineration process, the refractory bricks within the furnace must exhibit rapid heating capabilities to reduce the duration of the incineration cycle, thereby enhancing operational efficiency and reducing energy consumption. Additionally, superior heat dissipation properties allow for the rapid cooling of the refractory bricks after each incineration cycle, minimizing the time at which they remain at high temperatures; this, in turn, extends their service life and improves their operational efficiency. Therefore, the heating and cooling rates are critical indicators for evaluating the performance of refractory bricks.

**Figure 8:**
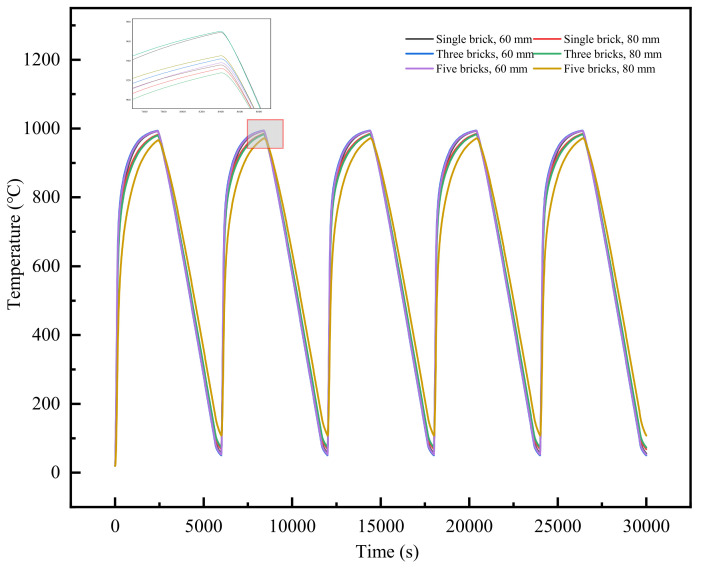
Temperature at the center of the refractory bricks’ recesses as a function of time.

The temperature at the center of the refractory bricks was analyzed. Figure 8 depicts the variation in the temperature with time for the coupled parameters of refractory brick number and brick thickness. It was observed that the temperature variation trends at the central point for the six scenarios were largely similar. For an equivalent number of refractory bricks, an increase in brick thickness led to a gradual decrease in both heating and cooling rates. Additionally, each thermal cycle exhibited a progressively lower peak temperature and a higher minimum temperature. However, when the brick thickness was held constant, the brick number had a minimal effect on temperature.

**Figure 9:**
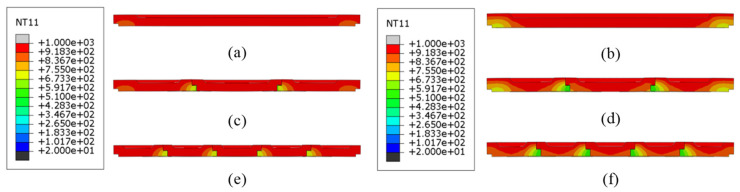
Temperature contour profiles of structures with different brick thicknesses and brick numbers. (**a**) Single brick, 60 mm; (**b**) single brick, 80 mm; (**c**) three bricks, 60 mm; (**d**) three bricks, 80 mm; (**e**) five bricks, 60 mm; and (**f**) five bricks, 80 mm.

Figure 9 illustrates the temperature field cloud profile of the refractory brick structure at the end of the maximum temperature in the first temperature cycle (T = 2400 s). Since the refractory brick model is a thin plate with a small thickness, the internal temperature distribution of the refractory brick cannot be observed from the three-dimensional temperature field map. Therefore, a temperature field profile was chosen to observe its internal temperature distribution. Additionally, it was observed that for refractory bricks of the same thickness, the internal temperature of the refractory bricks was almost the same at this point, but the temperature field was still weakly affected by the higher number of resistances. When the brick number was the same, a greater radial brick thickness led to a greater temperature gradient inside the refractory brick structure. This was because of Equation (1), which showed that a greater brick thickness leads to a longer heat transfer path and more time taken to conduct the heat. However, a comparison of the following profiles did not indicate that the thickness of the refractory bricks had a more significant effect on the temperature field of the refractory brick structure than the brick number.

#### 4.1.2. Displacement

**Figure 10:**
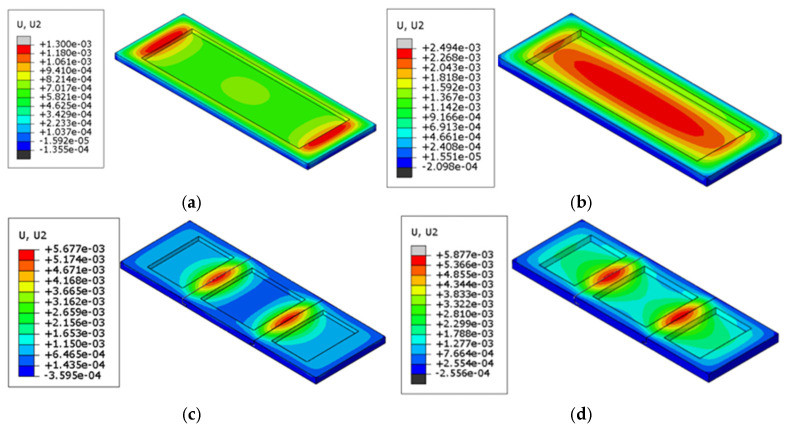

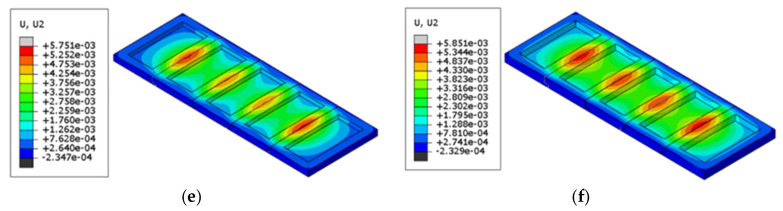
Points of maximum vertical displacement for corundum–silicon carbide refractory bricks. (**a**) Single brick, 60 mm; (**b**) single brick, 80 mm; (**c**) three bricks, 60 mm; (**d**) three bricks, 80 mm; (**e**) five bricks, 60 mm; (**f**) five bricks, 80 mm.

Table 4 presents the maximum vertical displacement of refractory brick structures with varying brick numbers and thicknesses after cyclic temperature loading. The vertical displacement was the largest for the model structure; hence, the data extraction focused on this aspect. Figure 10 illustrates the displacement contour lines at the maximum vertical displacement of the refractory brick structures. It can be observed from the figure that the three-brick and five-brick configurations exhibited greater deformation values compared to single refractory bricks, with the deformation values for the three-brick and five-brick configurations being relatively similar. It is also noteworthy that the maximum vertical displacement of the three-brick structure was not always larger than that of the five-brick structure. For instance, the three-brick structure with a radial thickness of 80 mm deformed by 5.88 mm, which was greater than the 5.85 mm deformation of the five-brick structure of the same thickness. For refractory brick structures with the same brick number, the maximum vertical displacement increased with an increase in thickness. The maximum difference in deformation between the refractory bricks of different numbers was 342.3%, and the maximum difference in deformation between refractory bricks of different thicknesses was 91.5%. Thus, the data directly indicate that the brick number had a greater impact on deformation than the brick thickness.

An interesting phenomenon was observed for the single-refractory-brick structures, where the position of maximum deformation shifted from the ends of the brick to the center as the brick thickness increased. In conjunction with the analysis from Figure 9, it is evident that a greater thickness of the refractory brick structure leads to a higher temperature gradient during the cooling process. More time is then required for heat to transfer from one end of the brick to the other, leading to the observed warping in thin bricks and arching in thick bricks. For multi-brick models, the maximum deformation was primarily located at the seams.

#### 4.1.3. Stress

**Figure 11:**
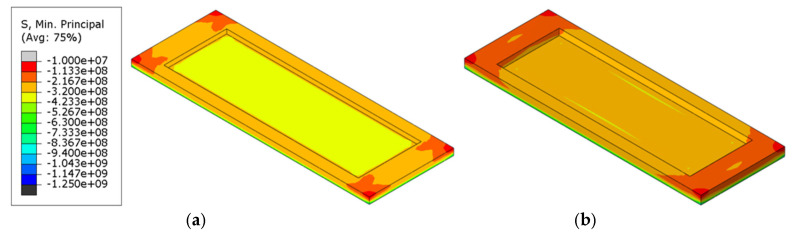

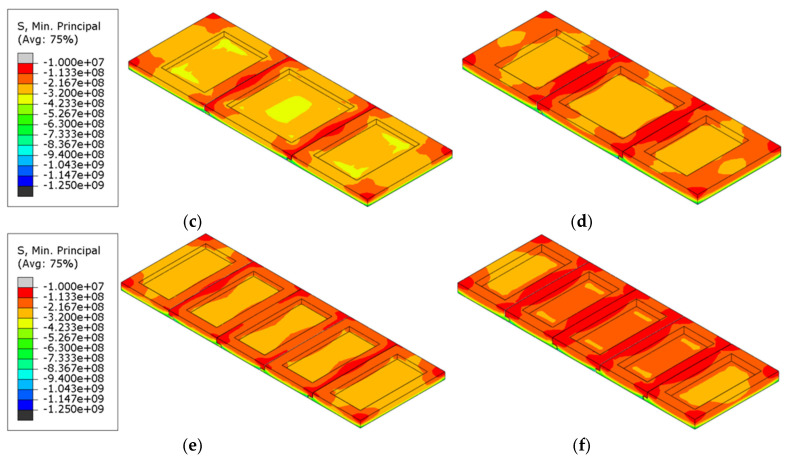
Maximum principal compressive stress cloud of refractory bricks (T = 2400 s). (**a**) Single brick, 60 mm; (**b**) single brick, 80 mm; (**c**) three bricks, 60 mm; (**d**) three bricks, 80 mm; (**e**) five bricks, 60 mm; and (**f**) five bricks, 80 mm.

Table 5 presents the magnitude of the maximum principal compressive stress under various refractory brick material and radial thickness coupling conditions. The difference in the maximum principal compressive stress due to varying numbers of bricks in the structure was 28.9%, while the difference due to varying brick thicknesses was 18.4%, suggesting that the brick number had a more significant impact on the stress distribution within the brick structure than the brick thickness. Figure 11 presents a cloud diagram of the maximum principal compressive stress at the conclusion of the first cycle to 1000 °C (T = 2400 s) and immediately after isothermal maintenance. An analysis of the figure indicated that stress was primarily concentrated at the corners of the refractory bricks or along their connecting seams. As shown, the distribution characteristics of the principal compressive stress in the refractory bricks were fundamentally similar, exhibiting higher stress in the central recess, lower stress at the edges, and the maximum value on the surface of the single-brick structures. The surface principal compressive stress on the brick structure gradually decreased with an increase in the brick number. This also indicated that an increased number of bricks introduced more seams, which, in turn, helped to distribute some of the principal compressive stress within the refractory brick structure.

### 4.2. Combined Effects of the Brick Material and Brick Thickness

Table 6 presents the coupling of different brick materials and radial thicknesses for three-brick corundum–silicon carbide refractory brick structures under high-temperature conditions at 1000 °C, forming six scenarios. A comparative study on the impact of these scenarios on the temperature, stress, and deformation of the refractory brick structure was conducted.

#### 4.2.1. Temperature

**Figure 12:**
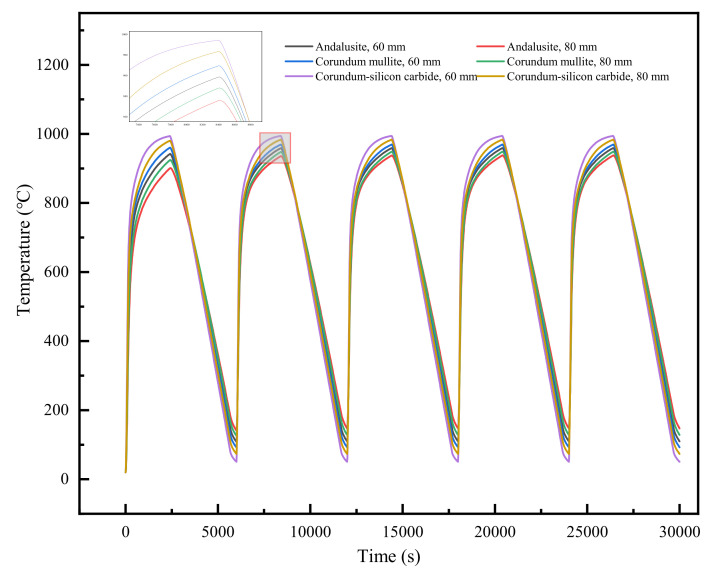
Temperature variation in the center of the refractory bricks’ recesses.

Figure 12 illustrates the temperature variation over time at the center of the recessed surface for refractory bricks under different coupling conditions of brick material and radial thickness. It was observed that there were certain differences in the temperature changes at the center for different scenarios. When the refractory brick models differed only in thickness, an increase in radial thickness resulted in a gradual decrease in heating and cooling rates, with the highest attainable temperature decreasing and the lowest temperature increasing. When the refractory brick models differed only in material, corundum–silicon carbide exhibited the highest maximum temperature and the lowest minimum temperature. Andalusite had the highest minimum temperature and the lowest maximum temperature. When the geometric structure of the refractory bricks was kept the same, these phenomena were consistent with the thermal conductivity values of the three materials presented in Table 2.

**Figure 13:**
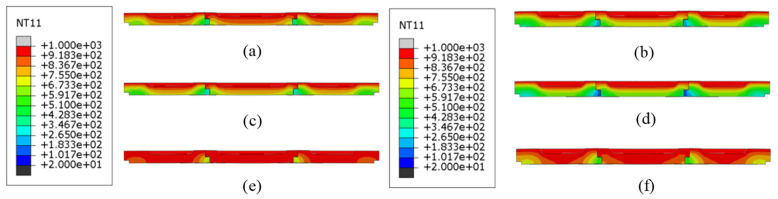
Temperature contour profiles of structures with different brick materials and brick thicknesses. (**a**) Corundum–mullite, 60 mm; (**b**) corundum–mullite, 80 mm; (**c**) andalusite, 60 mm; (**d**) andalusite, 80 mm; (**e**) corundum–silicon carbide, 60 mm; and (**f**) corundum–silicon carbide, 80 mm.

To further elucidate the impact of brick material and thickness on the temperature distribution within the refractory brick structure, Figure 13 depicts the temperature field contour of the refractory brick structure at the end of the highest temperature during the first temperature cycle (T = 2400 s). It is evident from the figure that for refractory bricks of the same material, a greater thickness leads to a larger vertical temperature gradient, indicating a more non-uniform temperature distribution within the brick structure. Among refractory bricks of different materials, the corundum–silicon carbide brick structure exhibited the smallest vertical temperature gradient and the most uniform temperature distribution, while the vertical temperature gradients of the andalusite and corundum–mullite brick structures were not significantly different from each other but were markedly higher than that of the corundum–silicon carbide brick structure. These differences in temperature gradients were primarily attributed to the distinct thermal conductivities of the materials, with corundum–silicon carbide having a markedly higher thermal conductivity than the other two. Additionally, it was observed from the temperature distribution diagram of the refractory brick structures that the influence of the brick material on the temperature distribution was significantly greater than that of the brick thickness.

#### 4.2.2. Displacement

**Figure 14:**
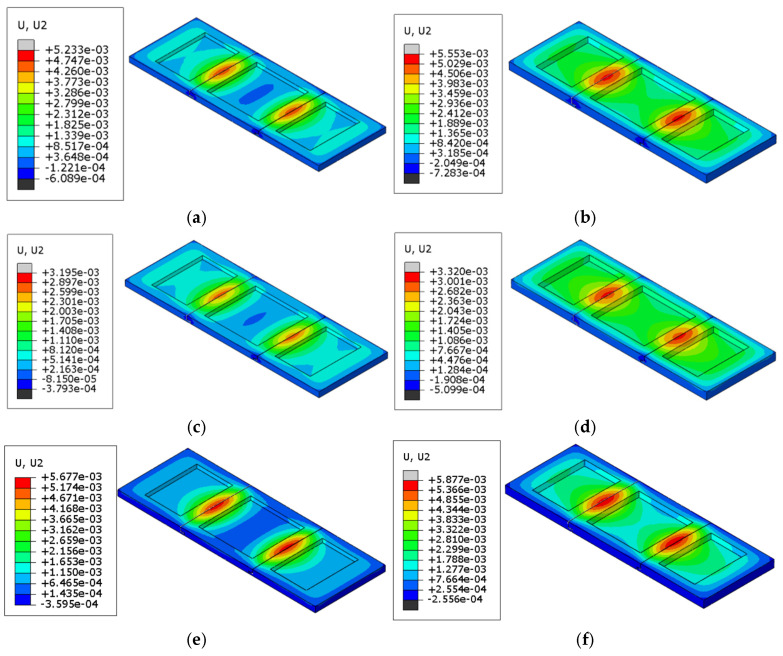
Points of maximum vertical displacement for refractory bricks (corundum–silicon carbide). (**a**) Corundum–mullite, 60 mm; (**b**) corundum–mullite, 80 mm; (**c**) andalusite, 60 mm; (**d**) andalusite, 80 mm; (**e**) corundum–silicon carbide, 60 mm; and (**f**) corundum–silicon carbide, 80 mm.

Table 7 depicts the maximum vertical displacement values for six different combi-nations of refractory brick materials and thicknesses. Figure 14 illustrates the vertical dis-placement distribution cloud diagrams for these various scenarios. It is evident that among refractory bricks of the same thickness, corundum–silicon carbide bricks experienced the greatest deformation, with both corundum–silicon carbide and corundum–mullite bricks showing significantly greater deformation than andalusite bricks. And the maximum difference in deformation was found to be 77.1%. When the bricks were of the same material, an increase in brick thickness did result in greater deformation, albeit to a lesser extent. Moreover, the maximum difference in deformation observed was 5.2%. It is clear from the following graphs that the maximum vertical displacement of the bricks was more affected by the type of brick material than by the brick thickness, aligning with the results previously discussed.

#### 4.2.3. Stress

**Figure 15:**
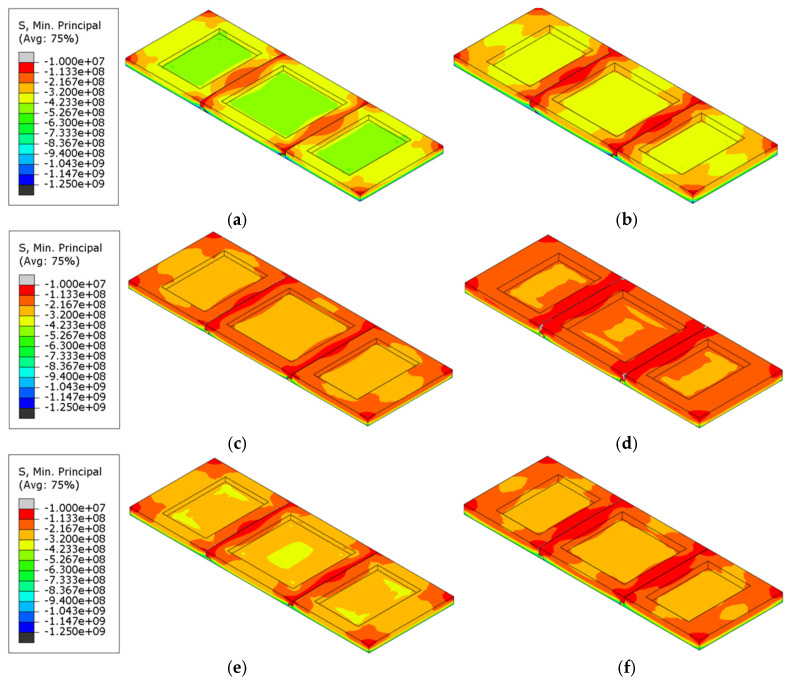
Maximum principal compressive stress cloud of refractory bricks (T = 2400 s). (**a**) Corundum–mullite, 60 mm; (**b**) corundum–mullite, 80 mm; (**c**) andalusite, 60 mm; (**d**) andalusite, 80 mm; (**e**) corundum–silicon carbide, 60 mm; and (**f**) corundum–silicon carbide, 80 mm.

Table 8 compares the impact of brick material and thickness on the maximum principal compressive stress across different refractory brick models. It was noted that among the different refractory brick materials, corundum–silicon carbide exhibited the highest maximum principal compressive stress, while andalusite showed the lowest. Both corundum–silicon carbide and corundum–mullite demonstrated significantly higher maximum principal compressive stresses than andalusite, with the greatest variation reaching 67.4%. For bricks of varying thicknesses, an increase in thickness corresponded to a higher maximum principal compressive stress, with the largest change being 7.8%. This suggests that the influence of brick material on stress within the brick structure was more significant than that of brick thickness. Figure 15 presents cloud diagrams of the maximum principal compressive stress for different brick models in the first temperature cycle (T = 2400 s), heated to 1000 °C. It is observable from the figure that the thickness had a relatively minor influence on the distribution of the maximum principal compressive stress, whereas the material had a distinct impact. The maximum principal compressive stress on the surface of corundum–mullite bricks was the greatest, and the minimum was observed for andalusite bricks.

### 4.3. Combined Effects of the Brick Number and Brick Material

Table 9 presents nine cases of refractory bricks with a radial thickness of 100 mm at 1000 °C involving different numbers of bricks and combinations of brick materials. In this section, a comparative study of the effects on the temperature distribution, stress, and deformation of these different refractory brick structures is presented.

#### 4.3.1. Temperature

**Figure 16:**
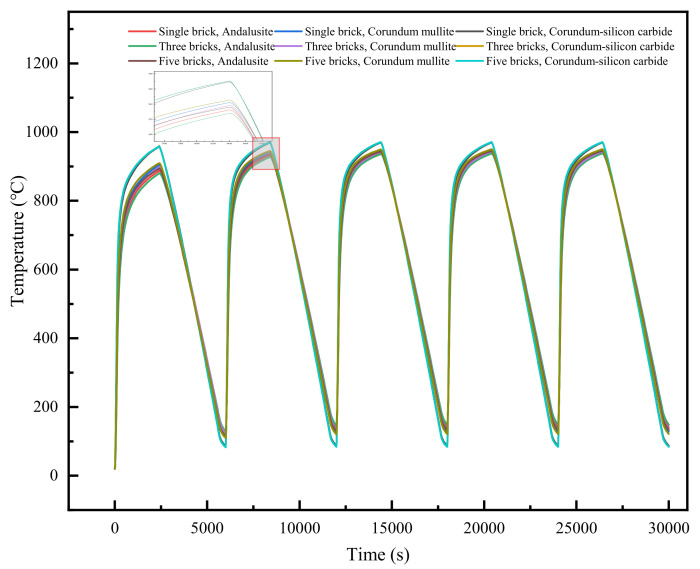
Temperature variation in the center of the refractory bricks’ recesses.

Figure 16 depicts how the temperature at the center of the recessed surface of 100 mm thick refractory bricks changed over time at 1000 °C for nine different scenarios with varying materials and numbers of bricks. It was found that when the brick number was identical and the brick material was corundum–silicon carbide, the refractory bricks experienced the fastest rate of temperature change. When the material was consistent, the rate of temperature change was similar regardless of the brick number. This was due to the bricks having the same thickness and heat transfer method. Furthermore, the material of the refractory bricks influenced the rate of temperature change more significantly than the brick number.

**Figure 17:**
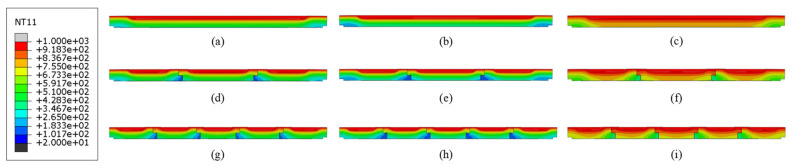
Temperature contour profiles of structures with different brick materials and brick numbers. (**a**) Single brick, corundum–mullite; (**b**) single brick, andalusite; (**c**) single brick, corundum–silicon carbide; (**d**) three bricks, corundum–mullite; (**e**) three bricks, andalusite; (**f**) three bricks, corundum–silicon carbide; (**g**) five bricks, corundum–mullite; (**h**) five bricks, andalusite; and (**i**) five bricks, corundum–silicon carbide.

Figure 17 illustrates the temperature field contour of the refractory brick structure at the end of the highest temperature within the first thermal cycle (T = 2400 s), showing the impact of the brick number and material on the temperature distribution. A vertical comparison revealed that for refractory bricks of the same material, the temperature distribution under high-temperature conditions did not significantly change with an increase in brick number, indicating that the brick number had little impact on the heat transfer capability of the structure. In contrast, a horizontal comparison showed that, among refractory brick structures with the same number of bricks, the corundum–silicon carbide brick structure exhibited the smallest temperature gradient and the most uniform temperature distribution, while corundum–mullite and andalusite refractory bricks shared similar characteristics. This is consistent with the conclusions drawn in Section 3.1 and Section 3.2, which attributed the differences to the distinct thermal conductivities of the various materials. The analysis, in conjunction with the figure, indicates that the influence of the brick number on the temperature distribution is less significant than that of the brick material. This phenomenon further underscores the importance of material selection when choosing refractory bricks.

#### 4.3.2. Displacement

**Figure 18:**
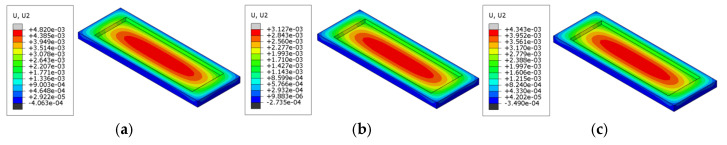

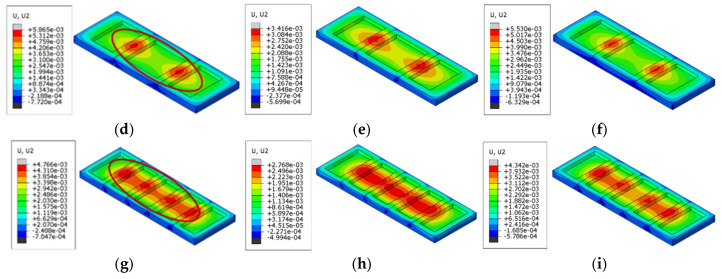
Points of maximum vertical displacement for refractory bricks (corundum-silicon carbide). (**a**) Single brick, corundum-mullite; (**b**) single brick, andalusite; (**c**) single brick, corundum-silicon carbide; (**d**) three bricks, corundum-mullite; (**e**) three bricks, andalusite; (**f**) three bricks, corundum-silicon carbide; (**g**) five bricks, corundum-mullite; (**h**) five bricks, andalusite; and (**i**) five bricks, corundum-silicon carbide.

Table 10 lists the maximum vertical displacement values of the refractory brick models under various coupling conditions of brick numbers and materials, which were compared with the cloud diagrams of the maximum vertical displacement distribution of the refractory brick structure depicted in Figure 18. The analysis revealed that, when the material of the refractory bricks was the same, the three-brick model exhibited the greatest deformation, while the five-brick model showed the least deformation, with a maximum difference of 27.4%. When the brick number was consistent, the corundum–mullite bricks had the greatest deformation, and the andalusite bricks had the least deformation, showing a maximum difference of 71.6%. It was worth noting that the andalusite refractory bricks exhibited minimal deformation, which was different from the deformation of the andalusite samples used in the validation experiments. This difference was due to the fact that in the refractory brick model, the bottom ring of the model was set up with a circular constraint and a downward load was applied to the upper surface of the refractory brick. At this time, due to the small elastic modulus of andalusite and the combined influence of the boundary conditions, the linear expansion coefficient of the material played a dominant role in the deformation with the temperature increase. Hence, the andalusite refractory bricks had the smallest amount of deformation. This indicated that the vertical deformation of the refractory bricks was more significantly influenced by the material than by brick number. In all cases, the maximum values of the vertical displacement cloud diagrams were concentrated in the central red ring area of the refractory bricks.

Furthermore, the cloud diagrams in Figure 11a,b, as well as Figure 18c, showed maximum deformation values of 1.30 mm, 2.49 mm, and 4.34 mm, respectively. It was observed that when the material and number of bricks were constant, the maximum vertical displacement increased with an increase in the thickness of the refractory bricks. Additionally, upon close examination of the vertical displacement cloud diagrams for the single-brick model structure, a trend was identified where the refractory brick structure deformed from warping to bulging, implying that the maximum vertical displacement values transitioned from the ends to the center of the refractory bricks. This phenomenon was attributed to the increased temperature gradient within the brick structure as the radial thickness increased, corroborating the conclusions drawn in the preceding text.

#### 4.3.3. Stress

**Figure 19:**
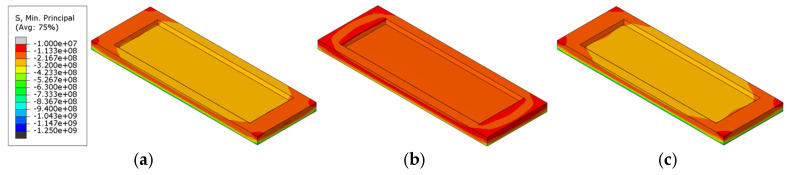

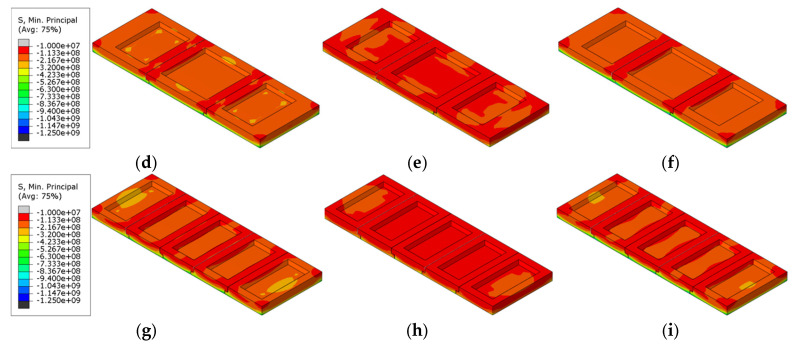
Maximum principal compressive stress cloud of refractory bricks (T = 2400 s). (**a**) Single brick, corundum-mullite; (**b**) single brick, andalusite; (**c**) single brick, corundum-silicon carbide; (**d**) three bricks, corundum-mullite; (**e**) three bricks, andalusite; (**f**) three bricks, corundum-silicon carbide; (**g**) five bricks, corundum-mullite; (**h**) five bricks, andalusite; and (**i**) five bricks, corundum-silicon carbide.

Table 11 displays the maximum principal compressive stress values for bricks under various combinations of materials and numbers of bricks. The research data indicate that among models of the same material, three-brick structures had the highest maximum main compressive stress, while the five-brick structures had the lowest maximum main compressive stress, with a maximum difference of 14.1%. When the brick number was consistent, the maximum principal compressive stress was significantly influenced by the material, with corundum-silicon carbide exhibiting the highest values and andalusite the lowest, showing a maximum difference of 65%. This suggests that the material of the refractory bricks played a more critical role in affecting the maximum principal compressive stress than the brick number. Figure 19 presents cloud diagrams of the maximum principal compressive stress for different brick models in the first temperature cycle (T = 2400 s), heated to 1000 °C. The diagrams more intuitively demonstrate the impact of brick number and brick materials on the variation in the maximum principal compressive stress, and they once again confirmed that the influence of the brick material was more pronounced.

## 5. Conclusions

This study pioneered the establishment of a fully coupled thermo-mechanical finite element model for refractory bricks used in incineration furnaces, which was subsequently validated through thermal expansion experiments. This research innovatively applied the refractory brick models used for the coupling of structural parameters and brick materials, analyzing their impact on the structural performance of the bricks. The findings of the study are summarized as follows:

(1) Combined effects of the brick number and brick thickness: The numerical simulations revealed that the brick number had an inconspicuous effect on the temperature field but significantly impacted the maximum vertical displacement and the maximum principal compressive stress, with the maximum difference in vertical displacement was 342.3% between different brick numbers and 91.5% between different brick thicknesses. The maximum difference in principal compressive stress between different brick numbers was 28.9%, and that between different thicknesses was 18.4%. The single-brick structures showed minimal deformation, while three-brick configurations exhibited greater deformation than five-brick ones. Increased brick numbers facilitated a greater release of maximum principal stress and reduced deformation.
(2) Combined effects of the brick material and brick thickness: The brick material had a more pronounced effect on the temperature change rate, temperature field distribution, displacement field, and stress field of the refractory brick structure, with the maximum difference in vertical displacement was 77.1% between different brick materials and 5.2% between different brick thicknesses. The maximum difference in principal compressive stress between different brick materials was 67.4%, and that between different thicknesses was 7.8%. With increased brick thickness, there was a rise in the temperature gradients and internal distribution within the structure, causing greater displacement and stress. Corundum-silicon carbide bricks, as opposed to andalusite bricks, showed a more uniform temperature distribution and greater vertical displacement, with andalusite exhibiting a lower maximum principal compressive stress.
(3) Combined effects of the brick number and brick material: The material of the bricks had a more significant impact on temperature field distribution, displacement field, and stress field than the brick number, with the maximum difference in vertical displacement was 27.4% between different brick materials and 71.6% between different brick numbers. The maximum difference in principal compressive stress between different brick materials was 14.1%, and that between different brick numbers was 65%. Therefore, it is recommended that when selecting refractory bricks, the material should be the primary consideration, followed by the brick number and, finally, the brick thickness.
(4) An interesting discovery: In single-brick structures, the maximum vertical displacement increased with brick thickness. A transition from warping to bulging in the refractory brick structures was observed, with the maximum displacement shifting from the ends to the center. This was due to an increase in the temperature gradients with the brick thickness.

## Figures and Tables

**Table 1:** Specific input parameters in the FEM of refractory bricks.

Factors	Material	Brick Number	Brick Thickness (mm)
Brick number and brick thickness	Corundum–silicon carbide	Single, three, and five	60, 80, and 100
Material and brick thickness	Corundum–mullite, andalusite, and corundum–silicon carbide	Three	60, 80, and 100
Brick number and material	Corundum–mullite, andalusite, and corundum–silicon carbide	Single, three, and five	100

**Table 2:** Parameters of refractory materials.

Items	Corundum–Mullite	Andalusite	Corundum–Silicon Carbide
Density (g·cm^−3^)	2.90	2.53	2.98
Young’s modulus (Pa)	42 × 10^9^	37 × 10^9^	45 × 10^9^
Poisson’s ratio	0.3	0.3	0.3
Specific heat [J/(kg·K)]	700	650	700
Thermal conductivity [W/(m·K)]	2.3	1.5	4.6
Thermal expansion coefficient (K^−1^)	6.3 × 10^−6^	4.3 × 10^−6^	5.5 × 10^−6^

**Table 3:** Comparison of brick numbers and brick thicknesses.

Number	Brick Number	Brick Thickness (mm)
1	Single brick	60
2	Single brick	80
3	Three bricks	60
4	Three bricks	80
5	Five bricks	60
6	Five bricks	80

**Table 4:** Comparison of the maximum values of vertical displacement for different model parameters.

Number	Model Parameters	Maximum Vertical Displacement (mm)
1	Single brick, 60 mm	1.30
2	Single brick, 80 mm	2.49
3	Three bricks, 60 mm	5.68
4	Three bricks, 80 mm	5.88
5	Five bricks, 60 mm	5.75
6	Five bricks, 80 mm	5.85

**Table 5:** Comparison of maximum values of principal compressive stress.

Number	Model Parameters	Maximum Principal Compressive Stress (MPa)
1	Single brick, 60 mm	895.8
2	Single brick, 80 mm	1061
3	Three bricks, 60 mm	1155
4	Three bricks, 80 mm	1243
5	Five bricks, 60 mm	1019
6	Five bricks, 80 mm	1064

**Table 6:** Comparison of brick materials and brick thicknesses.

Number	Material	Brick Thickness (mm)
1	Corundum–mullite	60
2	Corundum–mullite	80
3	Andalusite	60
4	Andalusite	80
5	Corundum–silicon carbide	60
6	Corundum–silicon carbide	80

**Table 7:** Comparison of the maximum vertical displacement values for different model parameters.

Number	Model Parameters	Maximum Vertical Displacement (mm)
1	Corundum–mullite, 60 mm	5.23
2	Corundum–mullite, 80 mm	5.55
3	Andalusite, 60 mm	3.20
4	Andalusite, 80 mm	3.32
5	Corundum–silicon carbide, 60 mm	5.68
6	Corundum–silicon carbide, 80 mm	5.88

**Table 8:** Comparison of maximum values of principal compressive stresses.

Number	Model Parameters	Maximum Principal Compressive Stress (MPa)
1	Corundum–mullite, 60 mm	1147
2	Corundum–mullite, 80 mm	1235
3	Andalusite, 60 mm	701.6
4	Andalusite, 80 mm	742.5
5	Corundum–silicon carbide, 60 mm	1155
6	Corundum–silicon carbide, 80 mm	1243

**Table 9:** Comparison of brick numbers and brick thicknesses.

Number	Brick Number	Material
1	Single brick	Corundum–mullite
2	Single brick	Andalusite
3	Single brick	Corundum–silicon carbide
4	Three bricks	Corundum–mullite
5	Three bricks	Andalusite
6	Three bricks	Corundum–silicon carbide
7	Five bricks	Corundum–mullite
8	Five bricks	Andalusite
9	Five bricks	Corundum–silicon carbide

**Table 10:** Comparison of maximum vertical displacement values for different model parameters.

Number	Model Parameters	Maximum Vertical Displacement (mm)
1	Single brick, corundum-mullite	4.82
2	Single brick, andalusite	3.13
3	Single brick, corundum-silicon carbide	4.34
4	Three bricks, corundum-mullite	5.87
5	Three bricks, andalusite	3.42
6	Three bricks, corundum-silicon carbide	5.53
7	Five bricks, corundum-mullite	4.77
8	Five bricks, andalusite	2.77
9	Five bricks, corundum-silicon carbide	4.34

**Table 11:** Comparison of maximum values of principal compressive stresses.

Number	Model Parameters	Maximum Principal Compressive Stress (MPa)
1	Single brick, corundum-mullite	1215
2	Single brick, andalusite	712.5
3	Single brick, corundum-silicon carbide	1213
4	Three bricks, corundum-mullite	1258
5	Three bricks, andalusite	773.5
6	Three bricks, corundum-silicon carbide	1276
7	Five bricks, corundum-mullite	1103
8	Five bricks, andalusite	661.8
9	Five bricks, corundum-silicon carbide	1118

## Data Availability

The original contributions presented in this study are included in the article. Further inquiries can be directed to the corresponding authors.
